# Adenovirus infection in children hospitalized with pneumonia in Guangzhou, China

**DOI:** 10.1111/irv.12782

**Published:** 2020-08-05

**Authors:** Lirong Zou, Lina Yi, Jianxiang Yu, Yinchao Song, Lijun Liang, Qianfang Guo, Xue Zhuang, Yunqiang Zhang, Min Kang, Jie Wu

**Affiliations:** ^1^ Guangdong Provincial Center for Diseases Control and prevention Guangzhou China; ^2^ Guangdong Provincial Institute of Public Health Guangdong Provincial Centre for Disease Control and Prevention Guangzhou China; ^3^ Southern Medical University Guangzhou China

**Keywords:** hospitalization, human adenovirus, pneumonia

## Abstract

**Background:**

HAdV infection can cause a variety of diseases. Although infections with HAdVs often are mild, life‐threatening respiratory disease can occur. Pneumonia is one of the more serious types of HAdV‐induced respiratory disease in children. In this study, we determined the prevalence and genotype of HAdVs among children hospitalized with pneumonia in Guangzhou, China.

**Methods:**

Nasopharyngeal swabs (NPSs) were collected from children hospitalized with pneumonia in Guangzhou, China, from January 2013 to June 2019. HAdVs were detected by real‐time polymerase chain reaction assay, and hexon, fiber, and penton gene were amplified and used for phylogenetic analysis. Epidemiological data were analyzed using SPSS16.0 software.

**Results and Conclusions:**

A total of 1778 children hospitalized with pneumonia were enrolled. The overall HAdV detection rate was 3.26%. And the yearly detection rate varied from around 2.5% in 2013‐2017 to around 6% in 2018‐2019. Children >5 years had the highest HAdV infection rate. 92.86% of HAdV sequences obtained in this study were belonged to species B, and no recombination was observed. HAdV‐B7 and HAdV‐B3 were the common types detected in the study period, with the predominant HAdV genotype shifted from HAdV‐B3 in 2015‐2016 to HAdV‐B7 in 2017‐2018. The discrepancies in HAdV detection rates in different study period and changes of HAdV predominant types over time highlighted the importance of continued surveillance.

## 1 INTRODUCTION

Human adenovirus (HAdV) is a non‐envelope and double‐stranded DNA virus.^1^ It belongs to the Mastadenovirus genus (Adenoviridae family). The virus was first identified by Rowe et al in 1953. In recent years, new HAdV genotypes are increasingly recognized through the use of phylogenetic analysis based on whole genomic sequences. There are at least 90 genotypes of HAdVs have been recognized that can be subdivided into seven species from A to G.^2^, ^3^ Most HAdVs circulated globally, but predominant types differ between countries or geographic regions, and change over time.^4^

HAdV infection can cause a variety of diseases, including bronchitis, pneumonia, conjunctivitis, gastroenteritis, and hemorrhagic cystitis. Of the 90 HAdV genotypes, 55 have been reported to cause human diseases.^5^ Previous study revealed that the clinical manifestation of HAdV infection is largely depended on the infecting HAdV genotype, with genotype D specifically associated with conjunctivitis, genotypes F and G with gastroenteritis, and genotypes B, C, and E with respiratory infections.^6^ Although infections with HAdVs often are mild to moderate, life‐threatening respiratory disease can occur in younger children, immunocompromised patients, and those with underlying chronic diseases.^2^ Pneumonia is one of the more serious types of HAdV‐induced respiratory disease in children, which can cause significant morbidity and mortality.^7^ And the severe cases are prone to develop long‐term respiratory complications such as post‐infectious bronchiolitis obliterans (PIBO) and bronchiectasis, with a risk up to 30%.^8^ Children aged 6 months to 5 years are at higher risk of developing pneumonia than those of other age groups. However, reports about HAdV infections in children hospitalized with pneumonia in China are limited. The aim of this study was to determine the prevalence and genotype of HAdVs among children hospitalized with pneumonia in Guangzhou, China.

## 2 MATERIALS AND METHODS

### 2.1 Patients and specimens

Nasopharyngeal swabs (NPSs) used in this study were collected from children (≤15 years) hospitalized with pneumonia in a tertiary maternal and child health care hospital in Guangzhou, China, from January 2013 to June 2019. Patients, who were suspected of having acute respiratory tract infections and were diagnosed with pneumonia by chest radiography, were enrolled in this study. NPSs were collected within 24 h after admission. Specimens were stored in a viral transport medium at −70°C until analysis.

### 2.2 HAdV and common respiratory virus detection

Total nucleic acid was extracted by the automated nucleic acid purification system EZ1 Advanced XL (Qiagen) using Mag MAX Total Nucleic Acid Isolation Kit (Qiagen) according to the manufacturer's instructions. The hexon gene fragment was amplified to screen HAdV infection by real‐time PCR. The primer set was as follows: HAdV‐F 5′‐ GGATGCTTCGGRGT ACCTSAGT ‐3′, HAdV‐R 5′‐CCCCAKAYTGAAGTAGGTGTTCTGT‐3′, and probe 5′‐FAM‐CCGGGTCTGGTGCAGTTCGCC‐3′‐TAMRA. The real‐time PCR conditions comprised an initial denaturation step at 95°C for 10 minutes, followed by 40 cycles of 95°C for 15 seconds and 65°C for 1 minute. For each specimen, assays for other common respiratory viruses (respiratory syncytial virus/RSV, influenza virus/Flu, parainfluenza virus/PIV, human enterovirus/rhinovirus/HRV, human metapneumovirus/MPV, human bocavirus/BOV, and human coronavirus/COV) were performed with a one‐step reverse transcription polymerase chain reaction (qRT‐PCR) as described in previous study.^9^

### 2.3 HAdV genotyping

For HAdV‐positive samples, the hexon, fiber, and penton gene were amplified by polymerase chain reaction (PCR) as described in a previous study.^10^ The primer pairs HVR‐F (5′‐CAGGATGCTTCGGAGTACCTGAG‐3′) and HVR‐R (5′‐TTTCTG AAGTTCCACTCGTAGGTGTA‐3′), fiber‐F (5′‐CCCTCTTCCCAACTCTGGTA‐3′) and fiber‐R/CR (5′‐GGGGAGGCAAAATAACTACTCG‐3′/ 5′‐GAGGTGGCAGG TTGAATACTAG‐3′), and penton‐F (5′‐CTATCAGAACGACCACAGCAACTT‐3′) and penton‐R (5′‐TCCCGTGATCTGTGAGAGCRG‐3′) were used, which would amplify a 1685 bp, a 1153/1519 bp, and a 1253 bp product in hexon, fiber, and penton gene, respectively. PCR was performed with the following thermal profile: 94°C 10 minutes, 34 cycles of 94°C 30 seconds, 52°C 30 seconds, and 72°C 2 minutes, and followed by final extension at 72°C 10 minutes. The PCR products were purified and sequenced using an ABI3730xl DNA Analyzer at IGE Biotech Co., Ltd. The HAdV sequences generated in this study have been submitted to GenBank.

### 2.4 Phylogenetic analysis

The HAdV sequences generated in this study were aligned with HAdV strain sequences available in GenBank. Multiple sequence alignment was performed using ClustalW, and alignments were minimally edited by hand using Aliview. For genotyping, maximum‐likelihood (ML) trees were estimated for hexon, penton, and fiber genes, respectively, by using IQ‐Tree (IQ‐TREE: A Fast and Effective Stochastic Algorithm for Estimating Maximum‐Likelihood Phylogenies). The phylogenetic trees and genotypes pattern for each Guangdong sequences were visualized by using ggtree (ggtree: An r Package for Visualization and Annotation of Phylogenetic Trees with Their Covariates and Other Associated Data’, Methods in Ecology and Evolution, 8:28‐36.).

### 2.5 Statistical analysis

Epidemiological data were analyzed by using the statistical product and service solutions (SPSS) 16.0 software. Continuous data were expressed as median (interquartile range [IQR]) if not normally distributed. HAdV detection rates among different year and different populations were compared by chi‐square test or Fisher's exact test, as appropriate. Mann‐Whitney *U* test was used when comparing continuous (numerical) variables without normal distribution between the two groups. All tests were calculated in a two‐tailed manner, and the statistical significance was set at *P* < .05 level.

## 3 RESULTS

### 3.1 Patient characteristics and HAdV detection

**Figure 1:**
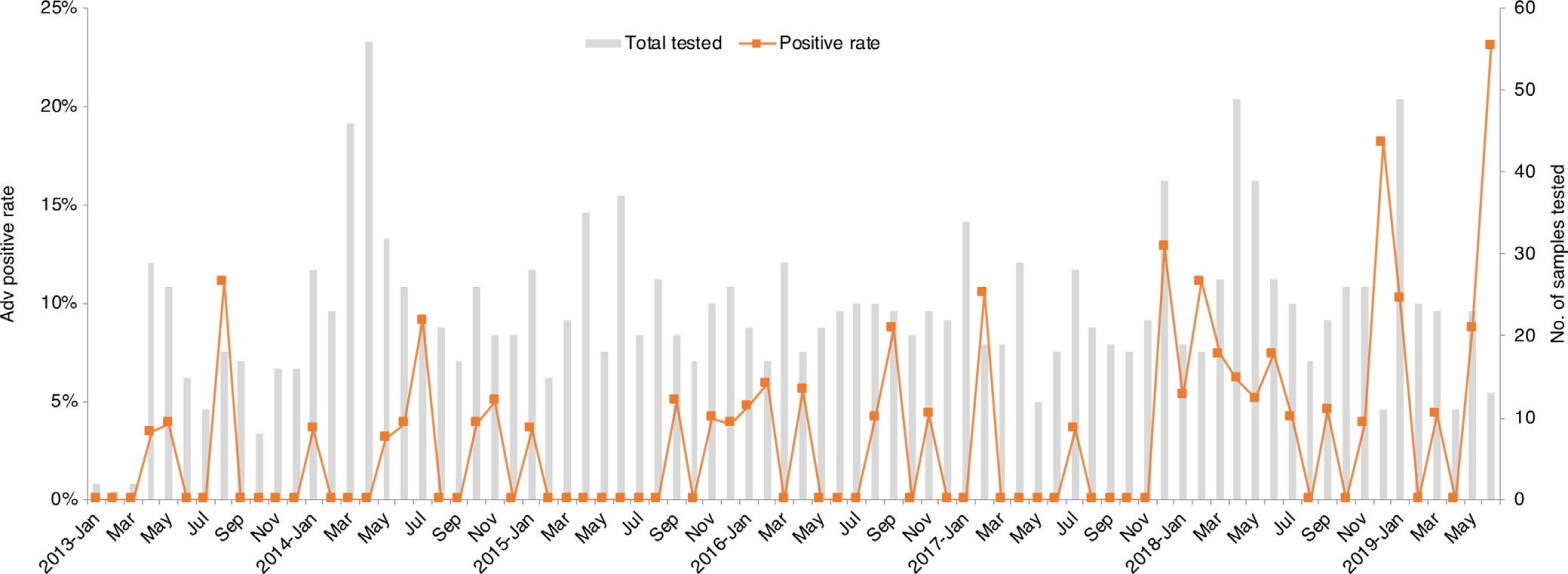
Distribution of HAdV‐positive cases among children hospitalized with pneumonia in Guangzhou, by month and year. Bars represent the total number of cases tested. The curve represents the HAdV‐positive rate for each month

A total of 1778 children hospitalized with pneumonia were enrolled during the study period (Table 1).These included 1154 males and 624 females (1.85:1). The median age was 11 months (interquartile range, 0.28 ~ 2.6 years), with more than half of the overall patients (60.5%) were children under 1 year of age. HAdV was detected in 58 samples, including 35 males and 23 females (1.52:1). No significant gender difference was observed (*P* = .459). The overall positive rate was 3.26%. The age of patients infected with HAdV varied from 3 months to 9 years with median age of 1 year and 9 months. Distribution of HAdV prevalence among age groups was significantly different (*P* = .000). The lowest detection rate was observed in age group of 1‐6 months (0.8%, 5/640), while the age group of more than 5 years old had the highest prevalence (6.5%, 11/170). We observed significant different positive rates of HAdV infection over the study years (*P* = .008, Figure 1). In year 2013‐2017, HAdV detection rate was approximately even, ranging from 1.4% to 2.9%. Then, an apparent increase of HAdV circulation in year 2018‐2019 was observed, with the positive rate up to 5.6% and 7.7%, respectively. HAdV infections circulated year‐round, with a tendency to be more common in the period from November of first year to January of the next, and a small peak between May and July.

**Table 1:** Demographic characteristics and HAdV detection among children hospitalized with pneumonia in Guangzhou between 2013 and 2019

Characteristic	Total tested	HAdV‐positive	*P* value
	n. % n = 1778	n. % n = 58	
Gender			
Male	1154, 64.90%	35, 60.34%	.459
Median age (interquartile range)	11 mo (0.28 ~ 2.6 y)	1.75 y (0.90 ~ 4.08 y)	.015
Age group			.000
≤0.5 y	640, 36.0%	5, 8.62%	
0.5‐1 y	436, 24.52%	20, 34.48%	
1‐3 y	369, 20.75%	16, 27.58%	
3‐5 y	163, 9.17%	6, 10.34%	
>5 y	170, 9.56%	11, 18.97%	
Year			.008
2013	161, 9.06%	4, 6.90%	
2014	337, 18.95%	7, 12.07%	
2015	289, 16.25%	4, 6.90%	
2016	265, 14.90%	7, 12.07%	
2017	278, 15.64%	8, 13.79%	
2018	305, 17.15%	17, 29.31%	
2019	143, 8.04%	11, 18.97%	

### 3.2 Co‐infections with other respiratory viruses

Of the 58 HAdV‐positive cases, 18 (31.03%) were co‐infected with other respiratory viruses (Table 2). Among which, 14 (77.78%) were with one other virus, 3 (18.75%) with two other viruses, and 1 (6.25%) with four other viruses. As shown in Table 2, HRV (37.50%) and PIV (31.25%) were the two most frequently identified viruses in co‐infected cases. No specific dominant genotype for HAdV was observed in co‐infections.

**Table 2:** Co‐detection of HAdV and other respiratory viruses

Co‐infection	Virus composition	Number of cases	HAdV genotype
2 viruses (N = 14)	HAdV + BOV	2	HAdV‐B3/ B7
	HAdV + HRV	2	HAdV‐B3/ B7
	HAdV + RSV	2	HAdV‐B3/ B7
	HAdV + PIV	3	HAdV‐B3/ B7/C1
	HAdV + COV	3	HAdV‐B3(2)/ B7
	HAdV + FluA	2	HAdV‐B7
3 viruses (N = 3)	HAdV + HRV+BOV	2	HAdV‐B3/ B7
	HAdV + HRV+PIV	1	HAdV‐B3
5 viruses (N = 1)	HAdV + HRV+PIV + COV+RSV	1	HAdV‐B7

### 3.3 Phylogenetic analysis and genotype distribution patterns

**Figure 2:**
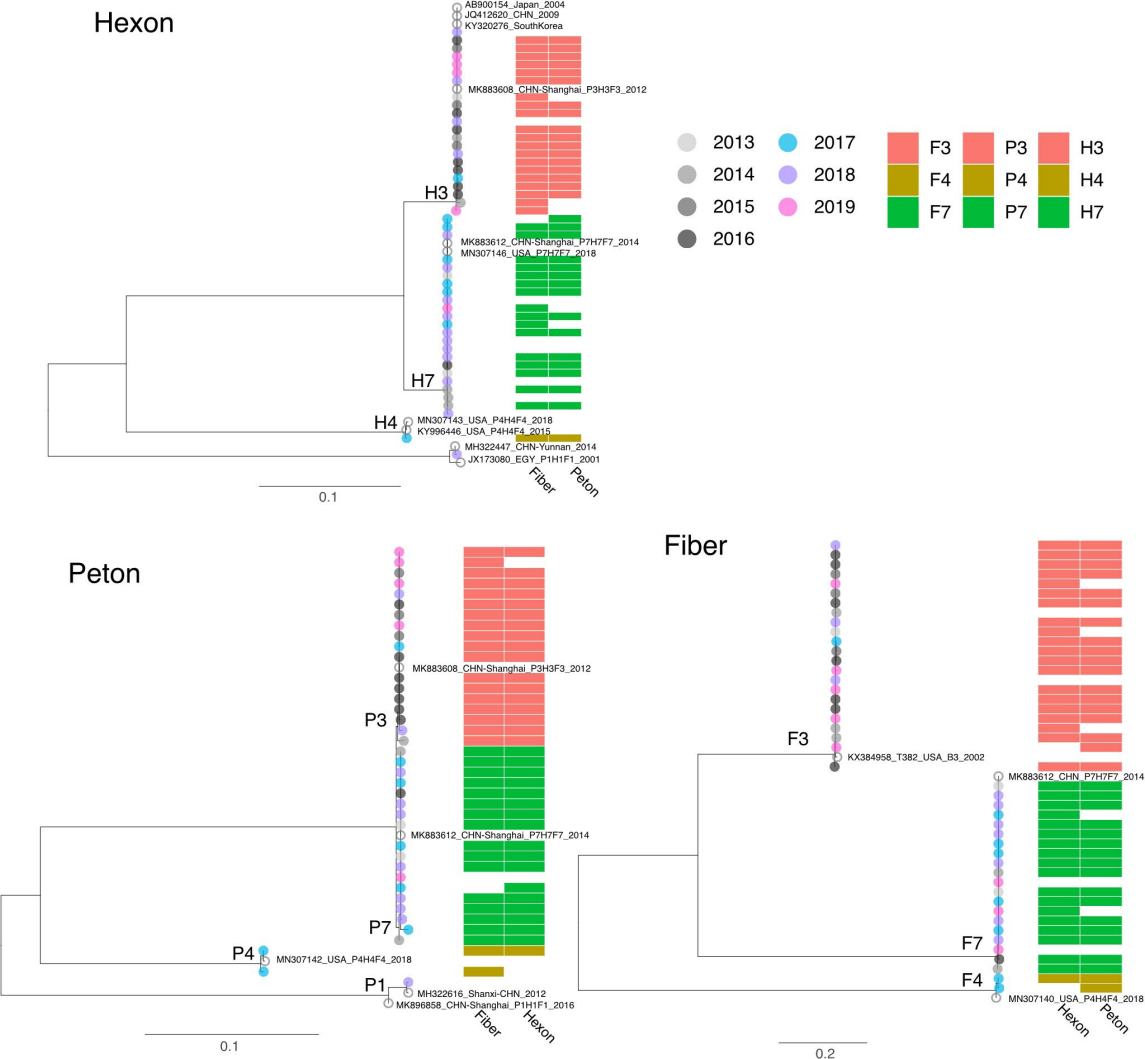
Phylogenetic analysis of HAdV strains identified in children hospitalized with pneumonia in Guangzhou between 2013 and 2019. The maximum‐likelihood tree was generated based on nucleic acid sequences of HAdV hexon, penton, and fiber gene, respectively. Guangzhou strains are represented in different colors depending on the year of sampling. Closely related reference strains (marked with circle) including in the phylogenetic tree were used to define the phylogenetic genotype of the gene. The corresponding genotypes of other two genes were shown on the right of each terminal branch

**Figure 3:**
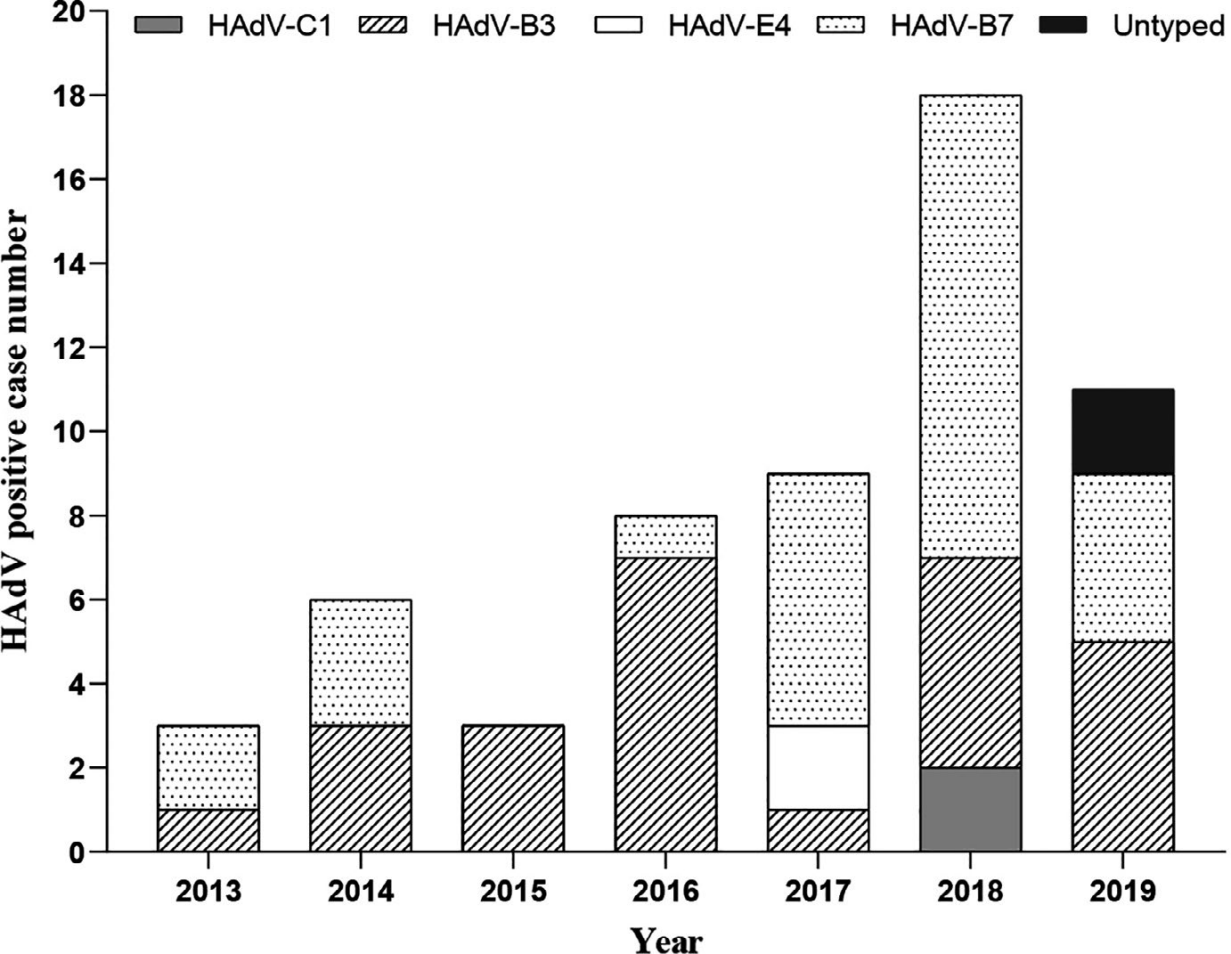
Yearly distribution of HAdV genotypes identified in children hospitalized with pneumonia in Guangzhou between 2013 and 2019

To further analyze the HAdV genotype, the hexon, fiber, and penton gene from HAdV positive specimens were amplified. In total, 56 out of 58 samples had at least one gene amplified. The hexon, fiber, and penton gene were successfully genotyped from 47, 45, and 39 samples, respectively. Consistent genotype distributions of three genes were observed for all Guangdong HAdV sequences, and no recombination was identified (Figure 2). The majority of HAdV strains were belonged to species B (52, 92.86%), except 4 HAdV stains which were distributed into species C (2) and species E (2), respectively. Of the 52 HAdV‐B strains, 27 were grouped with HAdV‐B7 (51.92%) and 25 were HAdV‐B3 (48.08%). The nucleotide sequence homologies of the hexon, fiber, and penton gene for HAdV‐B7 strains were between 99.9%‐100%, 99.8%‐100%, and 99.5%‐100%, respectively. For HAdV‐B3 strains, sequence analysis also showed high nucleotide sequence homologies (99.7%‐100%). All the HAdV‐B strains identified in this study were nearly identical to strains recently circulating in Shanghai Province, suggesting the genetic conservation of HAdV viruses in multiple regions of China (Figure 2). Yearly distribution of adenovirus serotypes in 2013‐2019 is shown in Figure 3. HAdV‐B3 and HAdV‐B7 strains were co‐circulated in the study period, except in year 2015 when only HAdV‐B3 was identified. The predominant HAdV genotype changed. We identified more HAdV‐B3 genotype in 2015‐2016, whereas HAdV‐B7 tends to be more common in 2017‐2018.

## 4 DISCUSSION

The present study reported the recent epidemiological profile of circulated HAdV strains in children hospitalized with pneumonia in Guangzhou China.

In this study, a total of 1778 samples were collected between January 2013 and June 2019. The overall positive rate of HAdV was 3.26%, which is consistent with prior reports conducted for hospitalized children with acute viral respiratory infection (1.70%‐13.90%).^11^ In China, HAdV‐positive rates vary from region to region, and it may change annually in the same area.^11^, ^12^, ^13^ Studies conducted in Beijing showed 20.1% of hospitalized children with SARIs were HAdV‐positive from 2007 to 2010.^13^ A lower detection rate has been reported in a recent study, and the HAdV was detected in 5.64% of the 1276 samples collected in Beijing in 2017‐2018.^11^ In Guangzhou, our previous data showed a proportion of 4.9% children with ARI were HAdV‐positive from 2006 to 2008.^14^ And in this study, the rates increased from around 2.5% in 2013‐2017 to around 6% in 2018‐2019. The discrepancies in HAdV detection rates in different study period and different areas highlighted the importance of continued surveillance. And factors such as targeted population, sampling period, and study duration should be considered when using results obtained in previous studies to assess recent virus burden in same geographic areas.

We did not observe any gender difference in HAdV infection, which is consistent with previous studies conducted in other provinces of China.^15^, ^16^ However, the predominance of male children was also commonly reported in literature.^8^ In our study, the highest HAdV‐positive rate was observed among children >5 years of age. This differs from previous reports that most HAdV infections occurred in early childhood due to the naive immune system.^17^ The age differences might be attributed to several factors. In our study, HAdV‐B7 was the most frequent isolated strains, and children with HAdV‐B7 infections tend to be older than those with HAdV‐B3.^18^ HAdV showed different seasonal patterns in different climatic zones. Previous studies demonstrated that the HAdV detection rates tended to coincide with higher temperature, longer sunshine duration, and lower wind speed.^11^ In our study, two peaks of HAdV were detected, maximal in May to July and November to January, consistent with those obtained in other subtropical areas.^8^

Reports about HAdV co‐infection with other respiratory viruses were frequent.^19^, ^20^, ^21^ In our study, co‐infection was identified in 18 out of 58 HAdV‐positive cases. Consistent with previous reports,^13^ the dominant co‐infection with HAdV was HRV and PIV. Because both HAdV‐B3 and HAdV‐B7 were commonly identified in single and co‐infections and no detailed clinical manifestations were recorded, it is difficult to associate co‐infections with specific HAdV types. And whether co‐infections of HAdV would present with more serious clinical symptoms was uncertain.

The majority (92.86%) of HAdV sequences obtained in this study were belonged to species B. Only four cases with HAdV‐E and C infection detected in 2017 and 2018, respectively. This was consistent with previous studies. Many studies have shown that HAdV respiratory infections are predominantly caused by species B (including types 3, 7, 11, 14, 21, and 55), C (types 1, 2, 5, and 6), and E (type 4).^22^ And compared with HAdV‐C and E species, HAdV‐B infections were more severe and significantly predominant in hospitalized patients. Of the 52 HAdV‐B strains that have been sequenced and analyzed, although HAdV‐B3 and HAdV‐B7 strains co‐circulated in the study period, the predominant HAdV genotype had shifted from HAdV‐B3 in 2015‐2016 to HAdV‐B7 in 2017‐2018. Genotypic composition observed in several other studies has demonstrated that the HAdV predominant types changed over time.^23^, ^24^ The HAdV epidemiology data collected in Taiwan showed that the HAdV‐B3 has circulated annually and was the dominant circulating genotype causing respiratory infections in children since 1999.^23^ An earlier study from our center also found that of the HAdV isolates from 2006 to 2008, 43.5% were HAdV‐B3.^14^ HAdV‐B7 only showed low prevalence before 2010, and has become the main types in China, Taiwan, and Singapore between 2011 and 2013.^25^, ^26^ It is possible that circulation of different subtypes may help these viruses to escape the pre‐existing immune response, and shifts in HAdV types might be associated with more infected cases. Different targeted population may also have different genotypic composition. Clinical features comparison between HAdV‐B3 and HAdV‐B7 showed that HAdV‐B3 was predominantly seen in children with upper respiratory tract infections while HAdV‐B7‐infected children were more likely to exhibit lower respiratory complications and required hospitalization.^24^, ^26^

This study has several limitations. First, as a retrospective study, no detailed clinical manifestations were recorded in our study. It is difficult to associate the specific clinical profiles with specific HAdV genotype. And whether co‐infections of HAdV would present with more serious clinical symptoms was uncertain. Second, although 56 HAdV‐positive specimens had at least one gene successfully amplified, only 33 samples have all three genes (hexon, fiber, and penton) successfully sequenced. HAdV serotype for 9 and 14 samples was based on 2 and 1 gene sequencing results, respectively. As recombination frequently shapes the evolution of HAdVs, information on a single gene may not sufficient to allow accurate and precise typing. Full‐length genome analysis would provide more genomic detail and help to understand potential recombination.

Our study analyzed the HAdV infections among children hospitalized with pneumonia in Guangzhou from 2013 to 2019. To our knowledge, this is the first report that describes the prevalence, age distribution, seasonality, and molecular epidemiology of HAdV infections among children hospitalized with pneumonia in Guangzhou. We found 3.26% of children hospitalized with pneumonia were caused by HAdV infection. And the yearly detection rate varied. Children >5 years had the highest HAdV infection rate. The majorities of HAdV sequences obtained in this study were belonged to species B. HAdV‐B7 and HAdV‐B3 were the common types detected, with the predominant genotype changed from HAdV‐B3 to HAdV‐B7. Our results will form the basis to determine the burden of HAdV infection. The discrepancies in HAdV detection rates in different study period and the changes of HAdV predominant types over time highlighted the importance of continued surveillance.

## CONFLICT OF INTEREST

The authors declare that they have no competing interests.

## AUTHOR CONTRIBUTION

**LiRong Zou:** Investigation (equal); Writing‐original draft (equal). **LINA YI:** Formal analysis (equal); Investigation (equal); Software (equal); Writing‐original draft (equal). **Yingchao Song:** Investigation (supporting). **Jianxiang Yu:** Investigation (supporting). **Lijun Liang:** Investigation (supporting). **Qianfang guo:** Investigation (supporting). **xue zhuang:** Investigation (supporting). **Yunqiang Zhang:** Investigation (supporting). **min kang:** Investigation (supporting). **Jie Wu:** Funding acquisition (equal); Project administration (equal).

## ETHICAL APPROVAL

This study was approved by the Ethics Review Committee of the Guangdong Provincial Center for Disease Control and Prevention. Respiratory samples were collected for public health purposes. Written consent was obtained from all patients or their guardian(s), and data were anonymized for analysis.
